# The efficacy of Epigallocatechin-3-gallate (green tea) in the treatment of Alzheimer’s disease: an overview of pre-clinical studies and translational perspectives in clinical practice

**DOI:** 10.1186/s13027-017-0145-6

**Published:** 2017-06-19

**Authors:** Marco Cascella, Sabrina Bimonte, Maria Rosaria Muzio, Vincenzo Schiavone, Arturo Cuomo

**Affiliations:** 1 Division of Anesthesia and Pain Medicine, Istituto Nazionale Tumori – IRCCS - “Fondazione G. Pascale”, Via Mariano Semmola, 80131 Naples, Italy; 2 Division of Infantile Neuropsychiatry, UOMI - Maternal and Infant Health, ASL NA 3 SUD, Torre del Greco, Via Marconi, 80059 Naples, Italy; 3 Division of Anesthesia and Intensive Care, Presidio Ospedaliero “Pineta Grande”, Castel Voltuno, 81100 Caserta, Italy

**Keywords:** (−) - Epigallocatechin-3-O-gallate (EGCG), Natural compound, Inflammation, Oxidative stress, Alzheimer’s disease

## Abstract

Alzheimer’s disease (AD) is a neurodegenerative disorder and the most common form of dementia characterized by cognitive and memory impairment. One of the mechanism involved in the pathogenesis of AD, is the oxidative stress being involved in AD‘s development and progression. In addition, several studies proved that chronic viral infections, mainly induced by Human herpesvirus 1 (HHV-1), Cytomegalovirus (CMV), Human herpesvirus 2 (HHV-2), and Hepatitis C virus (HCV) could be responsible for AD’s neuropathology. Despite the large amount of data regarding the pathogenesis of Alzheimer’s disease (AD), a very limited number of therapeutic drugs and/or pharmacological approaches, have been developed so far. It is important to underline that, in recent years, natural compounds, due their antioxidants and anti-inflammatory properties have been largely studied and identified as promising agents for the prevention and treatment of neurodegenerative diseases, including AD. The ester of epigallocatechin and gallic acid, (−)-Epigallocatechin-3-Gallate (EGCG), is the main and most significantly bioactive polyphenol found in solid green tea extract. Several studies showed that this compound has important anti-inflammatory and antiatherogenic properties as well as protective effects against neuronal damage and brain edema. To date, many studies regarding the potential effects of EGCG in AD’s treatment have been reported in literature. The purpose of this review is to summarize the in vitro and in vivo pre-clinical studies on the use of EGCG in the prevention and the treatment of AD as well as to offer new insights for translational perspectives into clinical practice.

## Background

The term dementia covers a large range of heterogeneous diseases at clinical and histopathological levels. Loss of memory and progressive dysfunctions of neuronal materials are features commonly present in patients with dementia and severely impairing theirs quality of life. Classically, dementia is defined as “syndrome caused by neurodegeneration” whereas Alzheimer’s disease (AD), is the most common type of dementia, accounting for an estimated 60 to 80% of cases. Although the exact pathophysiology of AD is still unclear, emerging evidence suggests that microglia-mediated neuroinflammatory responses play an important role in AD’s pathogenesis [1, 2]. In addition, several studies proved that chronic viral infections, mainly induced by Human herpesvirus 1 (HHV-1), Cytomegalovirus (CMV), Human herpesvirus 2 (HHV-2), and Hepatitis C virus (HCV) could be responsible for AD’s neuropathology. It is of note that microglia, resident immune cells of the central nervous system (CNS), are significantly involved in the neuroinflammation process. Current evidences on AD’s mechanism showed that the principal pathological features of AD are represented by the accumulation of soluble amyloid β peptide (Aβ) in the brain and the neurofibrillary tangles [3]. Aβ is considered an important neuroinflammatory stimulus for microglia. Moreover, it is of note that Aβ-dependent microglial activation induces neuronal injury due to the secretion of various pro-inflammatory molecules such as tumour necrosis factor-α (TNFα), interleukin (I) L-6, IL-1β, reactive oxygen species (ROS), and reactive nitrogen species (NOS) [4]. Neuroinflammation and oxidative stress processes are responsible for the impairments of neurovascular unit’s functions, leading to axonal demyelination, local hypoxia–ischemia, and reduced repair of white matter damages [5].

Despite the large amount of data published on the AD’s pathogenesis, a limited number of therapeutic drugs have been developed so far. Natural compounds have been identified as promising agents for the prevention and treatment of neurological disorders [6] and AD due their antioxidants and anti-inflammatory properties [7]. As a consequence, many investigations have been performed, with the aim of to evaluate the neuroprotective effects of nutraceuticals, such as resveratrol [8], curcumin [9], pinocembrin [10], caffeine [11], the combination of *Panax ginseng*, *Ginkgo biloba*, and *Crocus sativus* [12], and salvia triloba associated to *Piper nigrum* [13].

Catechins flavonoids are contained in Green tea extract (GTE) and are defined as the active components of green tea, accounting for its therapeutic properties. The ester of epigallocatechin and gallic acid, (−)-Epigallocatechin-3-Gallate [EGCG; (2R,3R)-5,7-dihydroxy-2-(3,4,5-trihydroxyphenyl)-3,4-dihydro-2H-1-benzopyran-3-yl 3,4,5-trihydroxybenzoate] (Pubchem CID: 65,064), represents the principal bioactive polyphenol in the solid GTE (65% catechin content). Several studies showed that EGCG has important anti-atherogenic and anti-inflammatory properties [14, 15] with potential neuroprotective effects against cerebrovascular diseases. For examples, *Ahn* et al. showed that EGCG was able to inhibit the production of TNFα-induced monocyte chemotactic protein-1 from vascular endothelial cells [16], whereas *Lee* et al. studied the protective effects of EGCG against brain edema and neuronal damage after unilateral cerebral ischemia in gerbils [17]. In addition, it has been proved that EGCG bypassed the blood–brain barrier (BBB) and to reach the functional parts of the brain [18]. Moreover, EGCG appears to be safe even when administered at relatively high dose. Indeed, as *Lee* et al. showed, an amount up to 6 mg/kg of EGCG can be used without any side effects [19].

**Fig. 1:**
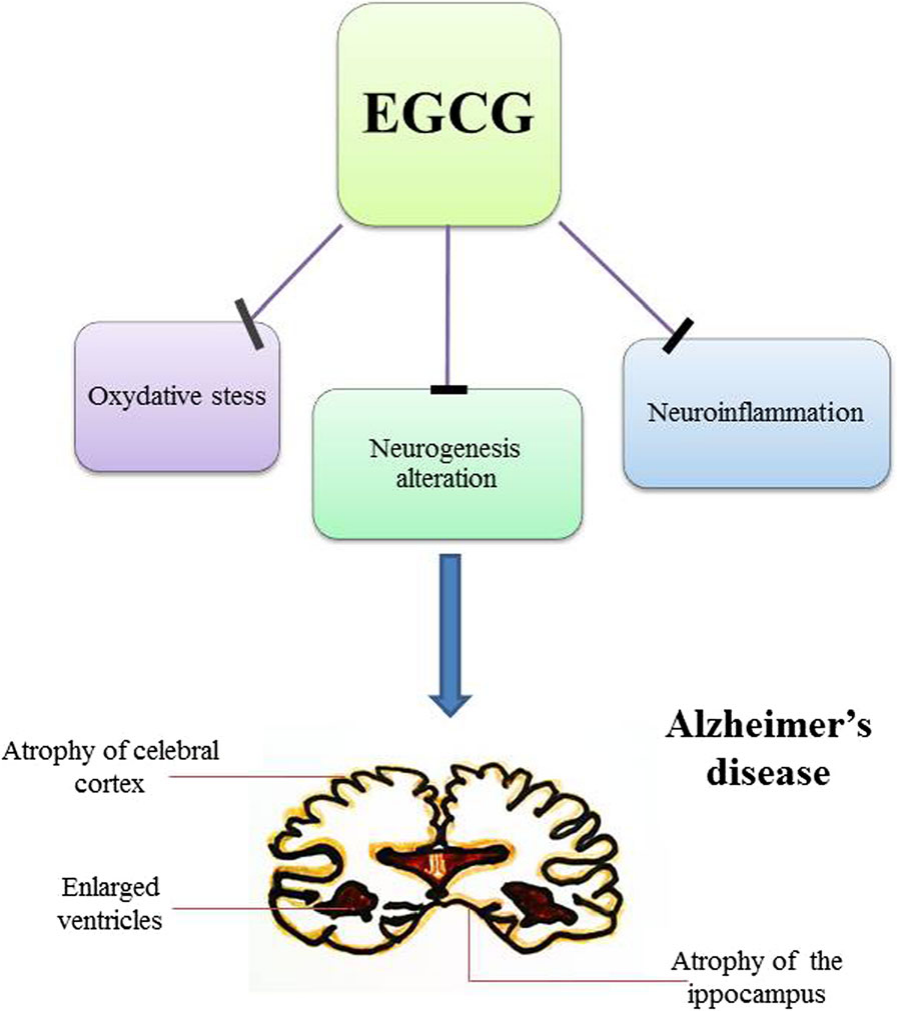
The potential effects of EGCG in Alzheimer’s pathogenesis

As regards to the role of EGCG in the treatment of AD’s disorder, a large amount of in vitro and in vivo studies, have been reported so far [19–37], indicating that EGCG plays a neuroprotective role and be potentially used as therapeutic agent for AD’s treatment (Fig. 1).

On the other side, studies have been also performed by using computational methods. For example, Ali et al., in order to demonstrate a potential role of cholinesterase inhibitors for AD’s treatment, performed an in silico analysis by using green tea polyphenols. Data emerged from this study, suggested that the cholinergic neurotransmission was enhanced by these synthetically compounds through the inhibition of acetylcholinesterase (AChE) and butyrylcholinesterase (BChE) enzymes [38].

The aim of this article is to highlight the potential use of EGCG for the prevention and/or treatment of AD, by summarize the pre-clinical studies reported in literature. Insights for translational perspectives into clinical practice are also given.

### Anti-neuroinflammatory properties of EGCG in the prevention and the treatment of Alzheimer’s disease

- **In vitro** ***studies: an update***
  In vitro studies on the anti-neuroinflammatory effects of EGCG have been performed on different cells lines including *MC65, EOC 13.31, SweAPP N2a, N2a/APP695, DIV8, CHO, and M146 L * cells (Table 1). Results from these studies showed that the anti-neuroinflammatory capacity of EGCG is mainly associated to the inhibition of microglia-induced cytotoxicity.
*Lin* et al. demonstrated that EGCG was able to suppress the neurotoxicity induced by Aβ, through the activation of the glycogen synthase kinase-3β (GSK-3β) and the inhibition of c-Abl/FE65 nuclear translocation [20]. This was a relevant finding, since it is of note that c-Abl is a cytoplasmic nonreceptor tyrosine kinase involved in the development of the nervous system and implicated in the regulation of cell apoptosis, whereas the β-isoform of GSK3 is a proline-directed serine-threonine kinase involved in neuronal cell development and energy metabolism [21]. These data suggest that c-Abl/GSK-3β signaling is involved in neuronal loss, neuroinflammation and gliosis. It is of note that, related to AD’s pathogenesis, the proteolytic processing of a transmembrane glycoprotein, known as amyloid precursor protein (APP), is responsible for the Aβ’s origin [22]. Other investigations have been conducted to evaluate the effect of EGCG on Aβ-induced inflammatory responses in microglia. To this regard, *Wei* et al.*,* investigated on the inhibitory effects of EGCG on microglial activation induced by Aβ and on neurotoxicity in Aβ-stimulated EOC 13.31 microglia. Results revealed that that EGCG was able to suppress the expression of TNFα, IL-1β, IL-6, and inducible nitric oxide synthase (iNOS) and to restore the levels of intracellular antioxidants against free radical-induced pro-inflammatory effects in microglia, the nuclear erythroid-2 related factor 2 (Nrf2) and the heme oxygenase-1 (HO-1) [23]. In addition, EGCG suppressed Aβ-induced cytotoxicity by reducing ROS-induced NF-κB activation and mitogen-activated protein kinase (MAPK) signaling, including c-Jun N-terminal kinase (JNK) and p38 signaling. Taken together these data suggest that EGCG is able to inhibit the neuroinflammatory response of microglia induced by Aβ and to protect against indirect neurotoxicity through several mechanisms.
  Previously, *Rezai-Zadeh* et al. demonstrated that GTE reduced the generation of Aβ in murine neuron-like cells (N2a) transfected with the human “Swedish” mutant APP (SweAPP N2a cells) and activated the nonamyloidogenic processing of APP, also promoting its α-secretase cleavage [24].
  One of the processes involved in the amyloid formation cascade, is the β-sheet formation. This event is frequently associated with cellular toxicity in many of human protein misfolding diseases, including AD. It has been reported that EGCG was able to interfere with this cascade, by redirection of prone polypeptides’ aggregation into off-pathway protein assemblies [25]. In another study, *Bieschke* et al. showed that EGCG converted the large mature Aβ fibrils into smaller forms with no toxicity for mammalian cell [26]. A recent study conducted by *Chesser* et al.*,* proved that EGCG in DIV8 primary rat cortical neurons, was able to enhance the clearance of AD-relevant phosphorylated tau species, indicating that EGCG could be used as an adjuvant agent for AD’s treatment [27]. Similar findings were obtained by *Chang* et al. The authors demonstrated that EGCG reduced β-amyloid (Aβ) accumulation in M146 L and CHO cells [28]. Finally, very recently it has been reported that EGCG inhibited β-Amyloid generation and oxidative stress involvement of nuclear receptor peroxisome proliferator-activated receptor gamma (PPARγ) in N2a/APP695 cells [29].
  All these data suggest that EGCG may be considered an important agent with neuroprotective properties against AD.
- **In vivo** ***preclinical studies: an-update***
  The neuroprotective effects of EGCG have been also demonstrated by in vivo experiments on several animal models (Table 2). The first study was reported by *Rezai-Zadeh* et al. [24]. The authors described that EGCG was able to decrease Aβ levels and plaques formation in a transgenic mouse model of “Swedish” mutant APP when was injected intraperitoneally (20 mg/kg). Similar results were obtained by the same group of researchers, when EGCG administered orally in drinking water (50 mg/kg), reduced Aβ deposition in the same mutant mice [30]. In another study based on the generation of transgenic mouse models of AD, *Li* et al.*,* [31] investigated on EGCG (orally 20 mg/kg/day, for 3 months) capacity to interfere with Aβ deposits in different brain areas. Data emerged by immunohistochemistry, showed that Aβ deposits were reduced by 60% in the frontal cortex and 52% in the hippocampus. A reduction of Thioflavine-S histochemistry labelling compact plaques was also detected in both regions. In addition, the percentage of CD45, a marker of microglial activation, was lower than 18% in the cortex and then 28% in the hippocampus respect to those observed in the control cohort. New insights on the role of EGCG in AD’s treatment have been reported by *Smith* et al. [32]. The authors engineered nanolipidic EGCG particles to improve oral’s bioavailability of EGCG. By using this system in mouse model of AD’s disease, the ability of EGCG for the treatment of AD was enhanced more than two-fold respect to treatment with free EGCG.
  The role of EGCG in AD’s treatment, was also described by *Giunta* et al.*,* in an interesting study in which was used Fish oil (8 mg/kg/day) combined to EGCG (62.5 mg/kg/day or 12.5 mg/kg/day) in a mouse model of AD. Results obtained from these experiments, showed that co-treatment of N2a cells with fish oil and EGCG increased the production of sAPP-alpha respect to either compound alone, indicating that these compounds were able to make a synergetic action on the inhibition of cerebral Aβ deposits [33].
  More recently*, Lee* et al. [19] tested the effects of EGCG on neuroinflammation and amyloidogenesis, in mice with systemic inflammation. The authors demonstrated that EGCG was able to prevent memory impairment induced by lipopolysaccharide (LPS) and apoptotic neuronal cell death. Moreover, EGCG prevented LPS-induced activation of astrocytes and increased cytokines expression (TNF-α, IL-1β, IL-6), suggesting that EGCG could be considered a therapeutic agent for neuroinflammation-associated AD. In animal another model of dementia, generated by infusion of streptozotocin (STZ) into intracerebroventricular (ICV) of rats, *Biasibetti* et al., showed that EGCG (10 mg/kg/day for 4 weeks) completely abrogated the cognitive deficit by influencing the glial-specific calcium binding protein S100B content in the hippocampus, the acetylcholinesterase (AChE) activity, the glutathione peroxidase activity, the nitric oxide (NO) metabolites, and ROS content [34].
*He* et al.*,* by using an AD’s mouse model generated by D-gal, showed that EGCG had a protective effect on AD by decreasing the expression of APP and Aβ in the hippocampus of mice [35]. Similarly, *Lin* et al. proved that EGCG impaired the formation of Aβ, through the inhibition of APP proteolysis, cAbl/FE65 complex nuclear translocation and GSK3 activation [20].
  Since insulin signaling plays a significant role in the regulation of synaptic activities involved in learning and memory processes, insulin resistance – expressed as increased phosphorylation levels of insulin receptor substrate-1 (IRS-1) - and subsequent alteration in signaling pathways, may contribute to the cognition impairment in AD’s patients [36]. Interestingly, *Jia* et al. demonstrated that EGCG reduced the spatial memory impairment (AD-related cognitive deficit) in APP/PS1 mice (bearing brain insulin resistance) by inducing IRS-1 signaling defects in the hippocampus, in a dose dependent manner [37].
  In another study, was reported that EGCG reduced Aβ accumulation in vitro and rescued cognitive deterioration in senescence-accelerated mice P8 (SAMP8). The authors showed that EGCG attenuated the cognitive deterioration in AD’s mouse model by upregulation of neprilysin (NEP) expression [28].
  Taken together, these data strongly suggest that EGCG could be used as a therapeutic agent for the treatment and the prevention of AD.

**Table 1:** A summary of in vitro studies on the role of EGCG on AD prevention

Cell lines	Drug and dosage	Results	References
*MC65*	EGCG 20 μM	EGCG reduced the Aβ levels by enhancing endogenous APP proteolysis and decreased nuclear translocation of c-Abl.	[20]
*EOC 13.31*	EGCG 5 to 20 μM	EGCG suppressed the expression of Aβ-induced TNFα, IL-1β, IL-6, and iNOS, and restored the levels of intracellular antioxidants Nrf2 and HO-1.	[23]
*SweAPP N2a*	GTE	EGCG reduced the Aβ generation and activated nonamyloidogenic processing of APP by promoting its α-secretase cleavage.	[24]
*Div8*	EGCG 12.5–50 μM	EGCG induced an increase in the key autophagy adaptor proteins NDP52 and p62.	[27]
*M146 L, CHO*	EGCG 16–32 μmol/L	EGCG reduced the accumulation of β-amyloid (Aβ).	[28]
*N2a/APP695*	EGCG (5–100 μM)	EGCG suppressed the production of Aβ and reduced inflammation, oxidative stress and cell apoptosis.	[29]

**Table 2:** Pre-clinical in vivo studies on the anti-neurodegenerative properties of EGCG in AD

Animal models	EGCG dose and route	Effects	Ref
*Tg2576 APP mice*	20 mg/kg daily for 4 months (oral gavage)	EGCG impaired Aβ formation by inhibiting APP proteolysis and by inhibiting cAbl/FE65 complex nuclear translocation and GSK3 activation.	[20]
*Swedish mutant APP-overexpressing mice (Tg APPsw line 2576)*	20 mg/kg (intraperitoneally)	EGCG induced APP processing with reduction of cerebral amyloidosis.	[24]
*APP transgenic mice*	20 mg/kg/day, for 3 months (oral gavage)	Aβ deposits were reduced by 60% in the frontal cortex and 52% in the hippocampus.	[31]
*AD mouse model*	Nanolipidic particles loaded with EGCG	Improved the bioavailability and α-secretase activity induced by EGCG.	[32]
*Tg2576 mice*	Fish oil (8 mg/kg/day) and EGCG (oral gavage, 62.5 mg/kg/day or 12.5 mg/kg/day)	Fish oil enhanced bioavailability of EGCG versus EGCG treatment alone. Synergetic effect of Fish oil and EGCG on the inhibition of cerebral A β deposits.	[33]
*Wistar rat model of dementia*	10 mg/kg/day for 4 weeks, oral gavage. ICV infusion of STZ (3 mg/kg)	EGCG completely abrogated the cognitive deficit, S100B content in the hippocampus, AChE activity, glutathione peroxidase activity, NO metabolites, and ROS content	[34]
*ICR mice model of systemic inflammation*	1.5 and 3 mg/kg for 3 weeks (Oral gavage). LPS (250 μg/kg) intraperitoneal	EGCG prevented LPS-induced memory impairment, apoptotic neuronal cell death, and microglia activation	[19]
*AD mouse model induced by D-gal*	2 mg/(kg/ day) or 6 mg/(kg/day) for 4 weeks, oral gavage	EGCG decreased the expression of APP and beta-Amyloid in the hippocampus of mice.	[35]
*APP/PS1 mice*	2 mg/(kg/day) or 6 mg/(kg/day) for 4 weeks, oral gave	EGCG treatment inhibited TNF-α/JNK signaling, increased the phosphorylation of Akt and glycogen synthase kinase-3β.	[37]
*SAMP8 mice*	5 and 15 mg/kg, for 60 days, intragastric	EGCG induced reduction in Aβ accumulation and increased NEP expression	[28]

### Translational perspectives of EGCG’s use into clinical practice

Promising results obtained *by* in vitro and in vivo studies on the use of EGCG as valuable therapeutic options for neurodegenerative disorders and cancer treatments, encourage its commitment to translation into clinical practice [39, 40].

- ***The bioavailability of EGCG in the brain: a discrepancy between animals and humans-based studies***
  Despite these encouraging results, there is still a translational gap between in vitro, in vivo and clinical studies with EGCG in neurodegenetative disease treatment. This can be associated to poor data reported on the bioavailability of EGCG in the brain, a feature extremely necessary for its neuroprotrective role. In vivo studies performed on animal models showed that repeated administration of EGCG, increased its accumulation in the brain [41, 42]. Opposite results were obtained in a study performed on six human subjects which assumed green tea by drinking. The products of green tea’s metabolism, (i.e. flavan-3-ol methyl-glucuronide and sulfate metabolites) were not able to reach the brain, thus remaining in the bloodstream [43]. These discrepancy can be associated to different causes, as reported by *Mähler* et al.in a review on this topic (i.e. dose, time point of EGCG treatment and different catechin metabolism between animals and humans) [39]. When EGCG is able to reach the brain, it regulates many biological processes and molecular signaling pathways involved in neurodegenerative disorders, included AD, as previously reported based on convincing results of in vitro and in vivo pre-clinical studies [44]. Unfortunately, these mechanisms are not completely elucidated in clinical studies probably due to the absence of standardization of disease severity in humans and in green tea preparations and its derivatives.
  This strongly suggest the needing of more detailed and specific studies on EGCG’s brain and plasma bioavailability, on its efficacy and safety in patients, and on possible interactions with other drugs.
- ***Clinical trials***
  Despite the encouraging data obtained from pre-clinical studies, several pivotal issues, regarding EGCG dose levels and administration frequency as well as genetic and epigenetic modulations involved in the metabolism and distribution of the active compounds in humans, remain to be explored [45].Previous findings from a cross-sectional study showed a negative association between green tea consumption and the prevalence of cognitive impairment in elderly individuals over 70 years old [46]. Thus, there is a discrepancy about the effects of GTE compounds on cognitive functions [47].
  Several clinical studies have been performed to evaluate the acute effects of EGCG and other constituents of tea, such as L-theanine, on cognitive function (e.g., attention) and mood. The results obtained, showed that tea consumption had significant acute benefits on mood and work performance and creativity [48, 49]. Another clinical study performed on 27 healthy human adults treated with EGCG (orally administered in a single dose of 135 mg), reported that EGCG was able to modulate cerebral blood flow parameters, without affecting cognitive performance or mood [50]. Similarly, *Scholey* et al. showed that EGCG administration (300 mg) was associated with reduced stress, increased calmness and increased electroencephalographic activity (increased alpha, beta and theta activities) in the midline frontal and central brain regions [51]. *Ide* et al. showed that green tea consumption in subjects with cognitive dysfunction (2 g/day for 3 months, approximately equal to 2 to 4 cups of tea/day) significantly improved cognitive performance [52].
  It is of note that the clinical symptoms of AD do not occur immediately. For this reasons, acute results of EGCG or other natural compounds on neurocognitive capacities, cannot be predictive of efficacy in more complex neurodegenerative diseases, such as AD’s syndrome. To date, one ongoing clinical trial is investigating on the effects of EGEG in early state of AD patients co-medicated with acetylcholine esterase inhibitors (ClinicalTrials.gov identifier: NCT00951834) [53].
  In order to evaluate the clinical effects of EGCG on AD, are necessary: (i) more detailed in vitro and in vitro studies with the purpose of dissect the underlying molecular mechanisms by which EGCG interferes with AD pathogenesis; (ii) clinical studies exploring the long-term effects of EGCG on cognitive functions; (iii) large size epidemiological studies concerning the consumption of EGCG and the progression of AD.

## Conclusions

Several in vitro and pre-clinical studies have demonstrated that EGCG, the principal bioactive component found in green tea, has anti-inflammatory properties by modulating different molecular pathways. Regarding AD’s syndrome, in vitro and in vivo studies reviewed here, showed that EGCG mainly induces reduction in Aβ accumulation, by modulating several biological mechanisms. Promising results in the pre-clinical and in recent clinical studies largely encourage EGCG’s commitment to translation into a clinical therapeutic approach. However, EGCG dose levels and administration frequency remain to be explored, so more pre-clinical investigations and well-drawn clinical trials are extremely needed.

With the use of different integrated approaches, next studies will shed light on the use of EGCG as a targeted prevention and individualized treatment to patients with AD’s disease.

## Abbreviations

AChE: Acetylcholinesterase
AD: Alzheimer’s disease
APP: Amyloid precursor protein
Aβ: Amyloid β peptide
BBB: Blood brain barrier
BChE: Butyrylcholinesterase
CNS: Central nervous system
EGCG: (−)-Epigallocatechin-3-O-gallate
GSK-3: glycogen synthase kinase-3
GTE: Green tea extract
HO-1: Heme oxygenase-1
ICV: Intracerebroventricular
IL: Interleukin
iNOS: Inducible nitric oxide synthase
IRS-1: Insulin receptor substrate-1
JNK: c-Jun N-terminal kinase
LPS: Lipopolysaccharide
MAPK: mitogen-activated protein kinase
NEP: Neprilysin
NO: Nitric oxide
NOS: Reactive nitrogen species
Nrf2: Nuclear erythroid-2 related factor 2
PPARγ: peroxisome proliferator-activated receptor gamma
ROS: Reactive oxygen species
S100B: Calcium-binding protein B
SAMP8: Senescence-accelerated mice P8
STZ: Streptozotocin
TNFα: Tumor necrosis factor-α
